# Supplementary material for: The impact of the caregiver mobility on child HIV care in the Manhiça District, Southern Mozambique: A clinical based study
Source: PLoS One. 2021 Dec 16;16(12):e0261356. doi: 10.1371/journal.pone.0261356 (PMC8675651; doi:10.1371/journal.pone.0261356)
Supplement: S1 File — (DOCX) [file pone.0261356.s001.docx]

|  | ***À preencher pelo acompanhante da criança na consulta TARV.*** |  |  |  |  |  |
| --- | --- | --- | --- | --- | --- | --- |
|  | **INFORMAÇÃO SOCIO-DEMOGRÁFICA** |  |  |  |  |  |
|  | **Número de estudo DIAC** \|__\|__\|__\|__\| |  |  |  |  |  |
|  | **Atualmente, a criança vive no Distrito da Manhiça**  **1=** Sim  **2=** Não  **2.1. Se o participante vive no DM, onde?**  **1=** Vila da Manhiça **5=** Maragra **9=** Palmeira/Nwamatibjana  **2=** Xinavane **6=** Maluana **10=** Taninga  **3=** Munguini **7=** Calanga **11=** 3 Fevereiro  **4=** Ilha Josina **8=** Xibukutsu **12=** Malavel  **14=** Outro \|__\|__\|__\|__\|__\|__\|__\|__\|__\|__\|__\| |  |  |  |  |  |
|  | **Com quem vive habitualmente a criança? (assinale todas aplicáveis)**  **1=** Mãe  **2=** Pai  **3=** Avó/ avô  **4=** Irmão/ irmã  **5=** Tio/ tia  **6=** Primo/ prima  **7=** Outro não familiar \|__\|__\|__\|__\|__\|__\|__\|__\|__\|__\|__\|__\|__\|__\|__\| |  | | | |  |
|  | **Quem é o cuidador primário da criança?**  **1=** Mãe  **2=** Pai  **3=** Avó/ avô  **4=** Irmão/ irmã  **5=** Tio/ tia  **6=** Primo/ prima  **7=** Outro não familiar \|__\|__\|__\|__\|__\|__\|__\|__\|__\|__\|__\|__\|__\|__\|__\| |  | | | |  |
|  | **Quem é o acompanhante da criança nesta consulta?**  **1=** Mãe  **2=** Pai  **3=** Avó/ avô  **4=** Irmão/ irmã  **5=** Tio/ tia  **6=** Primo/ prima  **7=** Outro não familiar \|__\|__\|__\|__\|__\|__\|__\|__\|__\|__\|__\|__\|__\|__\|__\| |  | | | |  |
|  | **O acompanhante da criança nesta consulta é cuidador primário ou cuidador acompanhante?**  **1=** Cuidador primário  **2=** Cuidador acompanhante |  | | | |  |
|  | **Onde vive a mãe da criança?**  1= No mesmo agregado que a criança  2= Num outro agregado dentro do distrito de Manhiça  3= Num outro agregado fora do distrito de Manhiça  4= Noutro pais  7.1. Se não vive com a criança, porque? (assinale todos os aplicaveis)  1= Faleceu  2= Migração ou viagens frequentes  3= Trabalho  4= Doença  5= Separação familiar  6= Abandono  7= Não quer responder  9= outro \|__\|__\|__\|__\|__\|__\|__\|__\|__\|__\|__\|__\|__\|__\|__\|  88= Não sabe |  | | | |  |
|  | **Onde vive o pai da criança?**  1= No mesmo agregado que a criança  2= Num outro agregado dentro do distrito de Manhiça  3= Num outro agregado fora do distrito de Manhiça  4= Noutro pais  8.1. Se não vive com a criança, porque? (assinale todas aplicáveis)  1= Faleceu  2= Migração ou viagens frequentes  3= Trabalho  4= Doença  5= Separação familiar  6= Abandono  7= Não quer respoder  9= outro \|__\|__\|__\|__\|__\|__\|__\|__\|__\|__\|__\|__\|__\|__\|__\|  88= Não sabe |  | | | |  |
|  | **O cuidador primário tem alguma situação que lhe obriga à dormir fora da sua casa por um período de pelo menos 4 noites seguidas durante a semana?**  1= Sim  2= Não  88= Não sabe Que tipo de situação? 1=mudança de casa  2=vive na Manhiça, com viagens frequentes    **9.2 A Mudança ou viagem foi dentro do distrito da Manhiça?**  **1=** Sim  **2=** Não    **9.3 Se foi fora do distrito da Manhiça, para onde?**  **1=** Outro distrito dentro de Moçambique  **2=** Outro país  **9.4. Se foi para outro distrito dentro de Moçambique, em que província?**  **1=** Maputo (cidade) **6=** Sofala  **11=** Cabo Delgado  **2=** Maputo Província **7=** Zambézia  **3=** Gaza  **8=** Nampula  **4=** Inhambane **9=** Tete  **5=** Manica **10=** Niassa  **12= Se não sabe, indique o nome do local onde vive** \|__\|__\|__\|__\|__\|__\|__\|__\|__\|__\|__\|  9.5. **Se foi para outro país, para qual?**  **1=** África do Sul  **2=** Suazilândia  **3=** Lesoto  **4=** Zimbabwe  **5=** Tanzânia  **6=** Botswana  **7=** Outro \|__\|__\|__\|__\|__\|__\|__\|__\|__\|__\|__\|__\|__\|__\|__\| |  | | | |  |
|  | **Nome completo** \|__\|__\|__\|__\|__\|__\|__\|__\|__\|__\|__\|__\|__\|__\|__\|__\|  \|__\|__\|__\|__\|__\|__\|__\|__\|__\|__\|__\|__\|__\|__\|__\|__\|  \|__\|__\|__\|__\|__\|__\|__\|__\|__\|__\|__\|__\|__\|__\|__\|__\| |  | | | |  |
|  | **Nome do pai** \|__\|__\|__\|__\|__\|__\|__\|__\|__\|__\|__\|__\|__\|__\|__\|__\|  \|__\|__\|__\|__\|__\|__\|__\|__\|__\|__\|__\|__\|__\|__\|__\|__\|  \|__\|__\|__\|__\|__\|__\|__\|__\|__\|__\|__\|__\|__\|__\|__\|__\| |  | | | | |
|  | **Nome da mãe** \|__\|__\|__\|__\|__\|__\|__\|__\|__\|__\|__\|__\|__\|__\|__\|__\|  \|__\|__\|__\|__\|__\|__\|__\|__\|__\|__\|__\|__\|__\|__\|__\|__\|  \|__\|__\|__\|__\|__\|__\|__\|__\|__\|__\|__\|__\|__\|__\|__\|__\| |  | | | | |
|  | **Nome Chefe agregado** \|__\|__\|__\|__\|__\|__\|__\|__\|__\|__\|__\|__\|__\|__\|__\|__\|  \|__\|__\|__\|__\|__\|__\|__\|__\|__\|__\|__\|__\|__\|__\|__\|__\|  \|__\|__\|__\|__\|__\|__\|__\|__\|__\|__\|__\|__\|__\|__\|__\|__\| |  | | | | |
|  | **Bairro em que vive** \|__\|__\|__\|__\|__\|__\|__\|__\|__\|__\|__\|__\|__\|__\|__\|__\| |  |  |  |  |  |
|  | **Data de nascimento** \|__\|__\| - \|__\|__\|__\| - \|__\|__\|__\|__\| |  | |  |  |  |
|  | **Perm_id do participante** \|__\|__\|__\|__\|- \|__\|__\|__\|-\|__\|__\| |  | | |  |  |
|  | **N. de HDD 02/009/**\|__\|__\|/\|__\|__\|__\|__\| |  | | |  |  |
|  | **Estado civil do cuidador primário:**   1. Solteiro (nunca viveu maritalmente) 2. Casado 3. União-de-facto 4. Divorciado 5. Separado 6. Viúvo |  | | |  |  |
|  | **Nível de escolaridade do cuidador primário:**   1. Não estudou 2. Abaixo de 5 ª classe 3. 5 ª classe 4. 7 ª classe 5. 10 ª classe 6. 12 ª classe 7. Ensino técnico elementar 8. Ensino técnico básico 9. Ensino técnico médio 10. Bacharel 11. Licenciatura 12. Mestrado 13. Doutoramento 14. Outro   **88=** Não sabe |  | | |  |  |
|  | **Qual é a principal fonte de rendimento do agregado onde vive o participante no distrito de Manhiça?**   1. Camponês 2. Assalariado 3. Sem salario fixo 4. Não sabe ou não quere responder 5. Outro \|__\|__\|__\|__\|__\|__\|__\|__\|__\|__\|__\| |  | | |  |  |
|  | **Religião do cuidador principal:**   1. Católica 2. Protestante/Anglicana 3. Cristão indeterminado 4. Islâmica 5. Hindus 6. Zione/ Sião 7. Animistas 8. Envangelica / pentecostal 9. Ateu 10. Outro (especifique) \|__\|__\|__\|__\|__\|__\|__\|__\|__\|__\|__\|   **88=** Não sabe  **99=** Recusa |  | | |  |  |
|  | **Quantos telefones celulares o cuidador primário tem?**  **1=** Nenhum  **2=** 1  **3=** 2-4  **4=** >5  **88=** nao sabe |  | | |  |  |
|  | **HISTÓRIA DE MIGRAÇAO** | |  |  |  |  |
|  | **Quantas vezes o cuidador primário se mudou de casa ou viajou para fora da Manhiça no último ano?**  1= 1 vez  2= 2 vezes  3= 3-5 vezes  4= uma vez por mês  5= uma vez por semana  6= outro \|__\|__\|__\|__\|__\|__\|__\|__\|__\|__\|__\|__\|__\|__\|__\|__\|  88= não sabe | |  |  |  |  |
|  | **Para onde mudou de casa ou viajou?**  1= Rural/ campo  2= Urbano/ cidade  3= Vários lugares de destino  88= Não sabe | |  |  |  |  |
|  | **O cuidador primário ficou por quanto tempo no lugar de destino?**  1= menos de 1 semana  2= menos de 15 dias  3= de 15 dias à 3 meses  4= de 3 à 12 meses  5= mais de 12 meses  88= não sabe | |  |  |  |  |
|  | **Quanto tempo fica em casa quando volta?**  **1=** Fim de semana  **2=** Menos de uma semana durante o meio de semana  **3=** De uma semana à um mês  **4=** De 1 a 3 meses  5= De 3 a 9 meses  6= Mais de 9 meses  **88=** Não sabe | |  |  |  |  |
|  | **A criança acompanhou ao cuidador primário quando este mudou de casa ou viajou?**  **1=** Sim  **2=** Não  **3=** Não sabe  27.1. Se Não, com quem ficou a criança?  **1=** Sozinha  **2=** Mãe  **3=** Pai  **4=** Avó/ avô  **5=** Irmão/ irmã  **6=** Tio/ tia  **7=** Primo/ prima  **8=** Outro não familiar \|__\|__\|__\|__\|__\|__\|__\|__\|__\|__\|__\|__\|__\|__\|__\| | |  |  |  |  |
|  | **Qual foi o motivo da vigem ou mudança de casa do cuidador primário?**  1= Trabalho  2= Estudos  3= Casamento ou festa  4= Falecimento  5= O cuidador esteve doente  6= Doença de um conhecido  7= Ajuda a uma outra pessoa (que não seja por doença)  8= Procura de melhores condições de vida  9= Outro \|__\|__\|__\|__\|__\|__\|__\|__\|__\|__\|__\|__\|__\|__\|__\|__\| | |  |  |  |  |
|  | **Que tipo de trabalho fez o cuidador primário no lugar de destino?**  1= Administrativo/ funcionário/ trabalho para o estado  2= Negocio próprio/ do patrão  3= Agricultura  4= Indústria  5= Mineiro  6= Vendedor  7= Trabalho doméstico  8= Não trabalha  9= Outro \|__\|__\|__\|__\|__\|__\|__\|__\|__\|__\|__\|  30.1 Se mineiro, em que província/ região trabalha?  \|__\|__\|__\|__\|__\|__\|__\|__\|__\|__\|__\|  30.2 Se mineiro, em que tipo de mina?  1= Ouro  2= Carvão  3= Platina  4= Diamante  5= Outro \|__\|__\|__\|__\|__\|__\|__\|__\|__\|__\|__\|  88= nao sabe  30.3 Se agricultor, em que província/ região trabalha?  \|__\|__\|__\|__\|__\|__\|__\|__\|__\|__\|__\| | |  |  |  |  |
|  | **Se o cuidador primário mudou de casa ou viajou para outro país, tem passaporte?**  1= Sim  2= Não  3= Não aplicável, nunca saiu de Moçambique  4= Não quer responder | |  |  |  |  |
|  | **O senhor/a senhora tem visto de trabalho?**  1= Sim  2= Não  3= Não precisa de visto  4= Outro \|__\|__\|__\|__\|__\|__\|  5= Não quer responder | |  |  |  |  |
|  | **De quem é a casa onde vive no lugar de destino?**  1= Casa familiar  2= Casa da empresa  3= Casa própria  4= Quarto/ casa alugado  5= Outro \|__\|__\|__\|__\|__\|__\|__\|__\|__\|__\|__\|__\|__\|__\| | |  |  |  |  |
|  | **Se o cuidador primário viajou para um outro pais, tem cartão de telefone celular no lugar de destino?**  1= Sim  2= Não  3= Não aplicável, não saiu de Mozambique  88= não sabe | |  |  |  |  |
|  | **O cuidador primário comunica-se através de telefone quando está no lugar de destino?**  1= Sim  2= não | |  |  |  |  |
| **HISTORIA HIV** | | |  |  |  |  |
|  | **Há quanto tempo foi diagnosticado de HIV?**  1= <3 meses 2= 3-12 meses 3=1-5 anos 4= > 5 anos 88= Não sabe  35.1. **Quando** **mudou de casa ou viajou já conhecia o seroestado de** HIV da criança?  **1=** Sim  **2=** Não  **88=** Não sabe  35.2. **Onde foi diagnosticado de HIV?**  1=Hospital Distrital da Manhiça  2= Outro Posto de Saúde dentro do distrito da Manhiça  3= Outro lugar dentro de Moçambique  4= Num outro país  88= Não sabe  35.3 **Se foi diagnosticado num outro pais, em qual?**  1= África do Sul  2= Suazilândia  3= Lesotho  4= Zimbabwe  5= Tanzânia  6= Botsuana  7= Outro \|__\|__\|__\|__\|__\|__\|__\|__\|__\|__\|__\|__\|__\|__\|__\|__\|  88= não sabe | |  |  |  |  |
|  | **Há quanto tempo a criança começou o TARV?**  1= <3 meses 2= 3-12 meses 3=1-5 anos 4= > 5 anos 88= Não sabe  **37.1.Quando** **mudou de casa ou viajou já estava a tinha iniciado TARV?**  **1=** Sim  **2=** Não  **88=** Não sabe |  |  |  |  |  |
|  | **A criança teve de aguardar pela autorização do cuidador primário para o início do TARV?**  **1=** Sim, do pai  **2=** Sim, da mãe  **3=** Sim, dum outro familiar  **4=** Não  **88=** Não sabe |  |  |  |  |  |
|  | **Desde que a criança iniciou seguimento nas consultas TARV, quantas vezes o cuidador primário mudou de casa ou viajou?**  1= 1 vez  2= 2 vezes  3= 3-5 vezes  4= uma vez por mês  5= uma vez por semana  6= outro \|__\|__\|__\|__\|__\|__\|__\|__\|__\|__\|__\|__\|__\|__\|__\|__\|  88= não sabe |  |  |  |  |  |
|  | Por quanto tempo esteve sem ir as consultas de seguimento de HIV?  1= 1-3 meses  2= 3-6 meses  3= 6-12 meses  4= > 1 ano  5= Não interrompeu o seguimento nas consultas de HIV  88= Não sabe  39.1. **Se interrompeu seguimento, indique o motivo (assinale todas aplicáveis)**  1= Esqueceu-se  2= Não teve um documento de transferência da criança  3= Não sabia a onde ir  4= O cuidador primário estava doente/ internado no hospital  5= A criança estava doente/ internado no hospital  6= indisponibilidade por trabalho  7= Problemas de transporte  8= Ausência do profissional de saúde no sevicio  9= mau atendimento  10= Efeitos secundários do tratamento  11= Esta em tratamento tradicional  12= Abandonou/ desistiu  13= Perdida de cartão  14= vergonha/ discriminação  15= Outro (especifique) \|__\|__\|__\|__\|__\|__\|__\|__\|__\|__\|__\|  88= não sabe |  |  |  |  |  |
|  | **Depois que o cuidador primário mudou de casa ou viajou, a criança continuou a vir as consultas de seguimento de HIV do Hospital Distrital da Manhiça?**  1= Sim, mensalmente  2=Sim, de 2 em 2 meses  3= sim de 3 em 3 meses  4=sim, de 6 em 6 meses  5= uma vez por ano  6= Não |  |  |  |  |  |
|  | **Se a criança também mudou de casa ou viajou, levou ou pediu guia de transferência para as consultas de seguimento de HIV no lugar de destino?**  **1=** Sim  **2=** Não  **3=** Não aplicável, a criança não viajou  **88=** Não sabe  41.1. Se não foi feita a guia de transferência, qual foi o motivo?  1= Não informou da mudança de residência  2= Pediu mas não foi aceite  3= outro \|__\|__\|__\|__\|__\|__\|__\|__\|__\|__\|__\|__\|__\|__\|__\|  88= não sabe |  |  |  |  |  |
|  | **Se a criança mudou de casa ou viajou, foi alguma vez as consultas de HIV no lugar de destino?**  1= Sim, alguma vez  2= Sim, regularmente  3= Não  88= Não sabe |  |  |  |  |  |
|  | **Com que frequência a criança assiste as consultas de seguimento de HIV? (seja no distrito de Manhiça ou num outro posto de saúde)**  1= Semanalmente 2= Mensalmente 3= De 3/3 meses 4= 6/6 meses 5= Uma vez por anos 88= Não sabe |  |  |  |  |  |
|  | **Se a criança mudou de casa ou viajou, teve acesso aos ARVs no lugar de destino?**  **1=** Sim  **2=** Não  **88=** Não sabe  44.1. Se não, qual foi o motivo?  **1=** Não procurou assistência clínica  **2=** Não sabia que poderia ser seguido no lugar de destino  **3=** Não foi aceite seguimento na unidade sanitária onde procurou  **4=** Não havia ARV  **5=** Outro \|__\|__\|__\|__\|__\|__\|__\|__\|__\|__\|__\|__\|__\|__\|__\|__\|__\|__\|__\|__\|__\|__\|__\|__\|__\|__\|  \|__\|__\|__\|__\|__\|__\|__\|__\|__\|__\|__\|__\|__\|__\|__\|__\|__\|__\|__\|__\|__\|__\|__\|__\|__\|__\|  **88=** Não sabe  44.2. Se sim, como teve acesso aos ARV no lugar de destino?  **1=** Enviados pela família/ conhecido  **2=** Farmácia local  **3=** Comprou no mercado ou loja  **4=** Outro \|__\|__\|__\|__\|__\|__\|__\|__\|__\|__\|__\|__\|__\|__\|__\|__\|__\|__\|__\|__\|__\|__\|__\|__\|__\|__\|__\| |  |  |  |  |  |
|  | **Durante a sua ausência, alguém levantou os ARV para a criança na farmácia do Hospital Distrital da Manhiça?**  **1=** Sim  **2=** Não  **3=** Não quer responder  **88=** Não sabe  45.1. Se alguém levantou os ARV para a criança, quem foi? (assinale todas aplicáveis)  **1=** A própria criança  **2=** Mãe  **3=** Pai  **4=** Avó/ avô  **5=** Irmão/ irmã  **6=** Tio/ tia  **7=** Primo/ prima  **8=** Outro não familiar \|__\|__\|__\|__\|__\|__\|__\|__\|__\|__\|__\|__\|__\|__\|__\|  45.2. Se alguém levantou para a criança, como chegaram à ela os ARV?  **1=** Correio  **2=** Alguém traz para o lugar de destino  **3=** O participante leva da Manhiça, quando regressa  **4=** Outro \|__\|__\|__\|__\|__\|__\|__\|__\|__\|__\|__\|__\|__\|__\|__\|__\|  45.3. Os ARV chegaram a tempo?  1=Sim, sempre  2=Sim, a maioria parte das vezes  3=Sim, as vezes (como metade/metade)  4= Não, nunca chegaram a tempo  **88=** Não sabe  45.4. O que costumava fazer quando a criança ficava sem ARV enviados da Manhiça?  **1=** Procurou os ARV na unidade sanitária do destino  **2=** Comprou os ARV no lugar de destino  **3=** Tomou menos comprimidos até os ARV chegar  **4=** Não fez tratamento até os ARV chegar  **5=** Outro \|__\|__\|__\|__\|__\|__\|__\|__\|__\|__\|__\|__\|__\|__\|__\|__\|__\|__\|__\|__\|__\|__\|__\|__\|__\|__\|  \|__\|__\|__\|__\|__\|__\|__\|__\|__\|__\|__\|__\|__\|__\|__\|__\|__\|__\|__\|__\|__\|__\|__\|__\|__\|__\|  \|__\|__\|__\|__\|__\|__\|__\|__\|__\|__\|__\|__\|__\|__\|__\|__\|__\|__\|__\|__\|__\|__\|__\|__\|__\|__\|  **6=** Não quer dizer |  |  |  |  |  |
|  | **Alguma vez a criança foi ao hospital por se encontrar doente desde foi diagnosticado HIV?**  **1=** Sim  **2=** Não  **88=** Não sabe |  |  |  |  |  |
|  | **Se a criança também mudou de casa ou viajou, alguma vez procurou assistência clínica no lugar de destino?**  **1=** Sim  **2=** Não  **3=** Não aplicável  **88=** Não sabe  **47.1.** Se sim, onde foi?  **1=** Urgências/Banco de Socorro  **2=** Consulta TARV  **3=** Farmácia  **4=** Triagem  **5=** Outro \|__\|__\|__\|__\|__\|__\|__\|__\|__\|__\|__\|__\|__\|__\|__\|__\|  **88=** Não sabe |  |  |  |  |  |
|  | **Alguma vez a criança esteve doente quando o cuidador primário estava fora?**  **1=** Sim  **2=** Não  **88=** Não sabe |  |  |  |  |  |
|  | **A criança alguma vez, esteve internada, desde o diagnóstico de HIV?**  **1=** Sim , na Manhiça  **2= sim, no lugar de destino**  **3=** Não  **88=** Não quer responder  49.1.**Se sim, quantas vezes esteve internado**? \|__\|__\| **88=** Não sabe |  |  |  |  |  |
|  | **Alguma vez a criança esteve internada quando o cuidador primário estava fora**, no ultimo ano?  **1=** Sim  **2=** Não  **88=** Não sabe |  |  |  |  |  |
|  | **Por quanto tempo esteve sem tomar os comprimidos contra o HIV (TARV)?**    1= 1-3 meses  2= 3-6 meses  3= 6-12 meses  4= > 1 ano  5= Não interrompeu o tratamento  88= Não sabe  51.1 Se interrompeu TARV, qual foi o motivo de interrupção?  **1=** Mudança de residência do cuidador primário  **2=** Doença do cuidador primário  **3=** Esqueceu  **4=** Cuidador primário sem dispensa no trabalho  **5= Sem medicamentos na farmácia do hospital**  **6=** Outro \|__\|__\|__\|__\|__\|__\|__\|__\|__\|__\|__\|__\|__\|__\|__\|__\|__\|__\|__\|__\|__\|__\|__\|__\|__\|__\|__\|  \|__\|__\|__\|__\|__\|__\|__\|__\|__\|__\|__\|__\|__\|__\|__\|__\|__\|__\|__\|__\|__\|__\|__\|__\|__\|__\|__\| |  |  |  |  |  |
|  | **Se a criança viajou, alguma vez fez seguimento de CD4 ou carga viral no lugar de destino?**  **1=** Sim  **2=** Não  **3=** não aplicável  **88=** Não sabe |  |  |  |  |  |
|  | **Se fosse possível, o cuidador primário gostaria de se poder comunicar com o Hospital Distrital da Manhiça por SMS no lugar de destino**  **1=** Sim  **2=** Não  **3=** não aplicável  **88=** Não sabe |  |  |  |  |  |
|  | **Se fosse possível, gostaria de poder levantar uma quantidade maior de ARV no Hospital Distrital da Manhiça para tomar no lugar de destino?**  **1=** Sim  **2=** Não  **88=** Não sabe Se Sim, gostaria de levar quantidade apara que período? 1= Apenas para 3 meses  2= 3-6 meses  3= Até 6 meses  4= outro (em meses) I_I_I_I |  |  |  |  |  |
|  | Qual das seguintes dificuldades encontrou quando regressou as consultas de seguimento de HIV no Hospital Distrital da Manhiça? ?  1. Muito tempo de espera 2. Mau atendimento por que não tinha cartão 3. Mau atendimento por que não se encontrava processo clinico 4. Mau atendimento por parte do pessoal 5. Não encontrou o local da consulta 6. Outros \|__\|__\|__\|__\|__\|__\|__\|__\|__\|__\|__\|__\|__\|__\|__\|__\| 7. Não teve dificuldades |  |  |  |  |  |
| **TFACTORES SOCIAIS** | |  |  |  |  |  |
|  | **A criança frequenta creche ou escola?**  **1=** Sim, regularmente  **2=** Sim, alguns dias  **3=** Não  **88=** Não sabe  **56.1. Se Sim, mantem a frequência à creche ou escola quando o seu cuidador está fora?**  **1=** Sim  **2=** Não  **88=** Não sabe  **56.2. Se sim, à que classe assiste a criança? \|__\|__\| classe** |  |  |  |  |  |
|  | **A criança tem o estado vacinal atualizado? (comprovar se o acompanhante tiver o cartão de saúde)**  **1=** Sim, todas  **2=** Sim, algumas vesses  **3=** Não  **88=** Não sabe |  |  |  |  |  |
