# Supplementary material for: The impact of the caregiver mobility on child HIV care in the Manhiça District, Southern Mozambique: A clinical based study
Source: PLoS One. 2021 Dec 16;16(12):e0261356. doi: 10.1371/journal.pone.0261356 (PMC8675651; doi:10.1371/journal.pone.0261356)
Supplement: S2 File — (DOCX) [file pone.0261356.s002.docx]

|  | ***À preencher pelo acompanhante da criança na consulta TARV.*** |  |  |  |  |  |
| --- | --- | --- | --- | --- | --- | --- |
|  | **INFORMAÇÃO SOCIO-DEMOGRÁFICA** |  |  |  |  |  |
|  | **Número de estudo DIAC** \|__\|__\|__\|__\| |  |  |  |  |  |
|  | **Atualmente, a criança vive no Distrito da Manhiça**  **1=** Sim  **2=** Não |  |  |  |  |  |
|  | **Se vive no Distrito da Manhiça, onde?**  **1=** Vila da Manhiça **5=** Maragra **9=** Palmeira/Nwamatibjana  **2=** Xinavane **6=** Maluana **10=** Taninga  **3=** Munguini **7=** Calanga **11=** 3 Fevereiro  **4=** Ilha Josina **8=** Xibukutsu **12=** Malavel  **14=** Outro \|__\|__\|__\|__\|__\|__\|__\|__\|__\|__\|__\| |  |  |  |  |  |
|  | **Com quem vive habitualmente a criança? (assinale todas aplicáveis)**  **1=** Mãe  **2=** Pai  **3=** Avó/ avô  **4=** Irmão/ irmã  **5=** Tio/ tia  **6=** Primo/ prima  **7=** Outro não familiar \|__\|__\|__\|__\|__\|__\|__\|__\|__\|__\|__\|__\|__\|__\|__\| |  | | | |  |
|  | **Quem é o cuidador da criança?**  **1=** Mãe  **2=** Pai  **3=** Avó/ avô  **4=** Irmão/ irmã  **5=** Tio/ tia  **6=** Primo/ prima  **7=** Outro não familiar \|__\|__\|__\|__\|__\|__\|__\|__\|__\|__\|__\|__\|__\|__\|__\| |  | | | |  |
|  | **Quem é o acompanhante da criança nesta consulta?**  **1=** Mãe  **2=** Pai  **3=** Avó/ avô  **4=** Irmão/ irmã  **5=** Tio/ tia  **6=** Primo/ prima  **7=** Outro não familiar \|__\|__\|__\|__\|__\|__\|__\|__\|__\|__\|__\|__\|__\|__\|__\| |  | | | |  |
|  | **O acompanhante da criança nesta consulta é cuidador primário ou cuidador acompanhante?**  **1=** Cuidador primário  **2=** Cuidador acompanhante |  | | | |  |
|  | **Onde vive a mãe da criança?**  1= No mesmo agregado que a criança  2= Num outro agregado dentro do distrito de Manhiça  3= Num outro agregado fora do distrito de Manhiça  4= Noutro pais  8.1. Se não vive com a criança, porque? (assinale todas aplicáveis)  1= Faleceu  2= Migração ou viagens frequentes  3= Trabalho  4= Doença  5= Separação familiar  6= Abandono  7= Não quer responder  9= Outro \|__\|__\|__\|__\|__\|__\|__\|__\|__\|__\|__\|__\|__\|__\|__\|  88= Não sabe |  | | | |  |
|  | **Onde vive o pai da criança?**  1= No mesmo agregado que a criança  2= Num outro agregado dentro do distrito de Manhiça  3= Num outro agregado fora do distrito de Manhiça  4= Noutro pais  9.1. Se não vive com a criança, porque? (assinale todas aplicáveis)  1= Faleceu  2= Migração ou viagens frequentes  3= Trabalho  4= Doença  5= Separação familiar  6= Abandono  7= Não quer responder  9= Outro \|__\|__\|__\|__\|__\|__\|__\|__\|__\|__\|__\|__\|__\|__\|__\|  88= Não sabe |  | | | |  |
|  | **Nome completo** \|__\|__\|__\|__\|__\|__\|__\|__\|__\|__\|__\|__\|__\|__\|__\|__\|  \|__\|__\|__\|__\|__\|__\|__\|__\|__\|__\|__\|__\|__\|__\|__\|__\|  \|__\|__\|__\|__\|__\|__\|__\|__\|__\|__\|__\|__\|__\|__\|__\|__\| |  | | | |  |
|  | **Nome do pai** \|__\|__\|__\|__\|__\|__\|__\|__\|__\|__\|__\|__\|__\|__\|__\|__\|  \|__\|__\|__\|__\|__\|__\|__\|__\|__\|__\|__\|__\|__\|__\|__\|__\|  \|__\|__\|__\|__\|__\|__\|__\|__\|__\|__\|__\|__\|__\|__\|__\|__\| |  | | | | |
|  | **Nome da mãe** \|__\|__\|__\|__\|__\|__\|__\|__\|__\|__\|__\|__\|__\|__\|__\|__\|  \|__\|__\|__\|__\|__\|__\|__\|__\|__\|__\|__\|__\|__\|__\|__\|__\|  \|__\|__\|__\|__\|__\|__\|__\|__\|__\|__\|__\|__\|__\|__\|__\|__\| |  | | | | |
|  | **Nome Chefe agregado** \|__\|__\|__\|__\|__\|__\|__\|__\|__\|__\|__\|__\|__\|__\|__\|__\|  \|__\|__\|__\|__\|__\|__\|__\|__\|__\|__\|__\|__\|__\|__\|__\|__\|  \|__\|__\|__\|__\|__\|__\|__\|__\|__\|__\|__\|__\|__\|__\|__\|__\| |  | | | | |
|  | **Bairro em que vive** \|__\|__\|__\|__\|__\|__\|__\|__\|__\|__\|__\|__\|__\|__\|__\|__\| |  |  |  |  |  |
|  | **Data de nascimento** \|__\|__\| - \|__\|__\|__\| - \|__\|__\|__\|__\| |  | |  |  |  |
|  | **Perm_id do participante** \|__\|__\|__\|__\|- \|__\|__\|__\|-\|__\|__\| |  | | |  |  |
|  | **N. de HDD 002/09/**\|__\|__\|**/** \|__\|__\|__\|__\| |  | | |  |  |
|  | **Nível de escolaridade do cuidador primário:**   1. Não estudou 2. Abaixo de 5ª classe 3. 5ª classe 4. 7ª classe 5. 10ª classe 6. 12ª classe 7. Ensino técnico elementar 8. Ensino técnico básico 9. Ensino técnico médio 10. Bacharel 11. Licenciatura 12. Mestrado 13. Doutoramento 14. Outro   **88=** Não sabe |  | | |  |  |
|  | **Qual é a principal fonte de rendimento do agregado onde vive o participante no distrito de Manhiça?**   1. Assalariado 2. Sem salario fixo 3. Vive de ajuda 4. Não sabe ou não quere responder 5. Outro \|__\|__\|__\|__\|__\|__\|__\|__\|__\|__\|__\| |  | | |  |  |
|  | **Trabalho do cuidador primário da criança:**  1= Administrativo/ funcionário/ trabalho para o estado  2= Negocio próprio/ do patrão  3= Agricultura  4= Indústria  5= Minero  6= Vendedor  7= Trabalho doméstico  8= Não trabalha  9= Outro \|__\|__\|__\|__\|__\|__\|__\|__\|__\|__\|__\|  88= não sabe |  | | |  |  |
|  | **Religião do cuidador primário:**   1. Católica 2. Protestante/Anglicana 3. Cristão 4. Islâmica 5. Hindus 6. Zione/ Sião 7. Animistas 8. Envangelica / pentecostal 9. Ateu 10. Outro (especifique) \|__\|__\|__\|__\|__\|__\|__\|__\|__\|__\|__\|   **88=** Não sabe  **99=** Não quer responder |  | | |  |  |
|  | **Quantos telemóveis celulares o cuidador primario tem?**  **1=** Nenhum  **2=** 1  **3=** 2-4  **4=** >5  **88=** nao sabe |  | | |  |  |
| **HISTORIA HIV** | | |  |  |  |  |
|  | **Há quanto tempo a criança foi diagnosticado de HIV?**  1= <3 meses 2= 3-12 meses 3=1-5 anos 4= > 5 anos 88= Não sabe  **23.2. Onde foi diagnosticada a criança?**  1= Hospital Distrital Manhiça  2= Outro Posto de saúde dentro do distrito de Manhiça  3= Outro lugar dentro de Moçambique  4= Num outro pais  88= Não sabe  **23.3 Se a criança foi diagnosticada num outro pais, em qual?**  1= África do Sul  2= Suazilândia  3= Lesotho  4= Zimbabwe  5= Tanzânia  6= Botsuana  7= Outro | |  |  |  |  |
|  | **Há quanto tempo a criança começou o TARV?**  1= <3 meses 2= 3-12 meses 3=1-5 anos 4= > 5 anos 88= Não sabe |  |  |  |  |  |
|  | **Com que frequência a criança atende as consultas de seguimento de HIV?**  1= Semanalmente  2= Mensalmente  3= De 3/3 meses  4= 6/6 meses  5= Uma vez por anos  88= Não sabe |  |  |  |  |  |
|  | Por quanto tempo esteve sem ir as consultas de seguimento de HIV?  1= 1-3 meses  2= 3-6 meses  3= 6-12 meses  4= > 1 ano  5= Não interrompeu o seguimento nas consultas de HIV  88= Não sabe  26.1. **Se interrompeu seguimento, indique o motivo (assinale todas aplicáveis)**  1= Esqueceu-se  2= Não teve um documento de transferência da criança  3= Não sabia a onde ir  4= O cuidador primário estava doente/ internado no hospital  5= A criança estava doente/ internado no hospital  6= indisponibilidade por trabalho  7= Problemas de transporte  8= Ausência do profissional de saúde no sevicio  9= mau atendimento  10= Efeitos secundários do tratamento  11= Esta em tratamento tradicional  12= Abandonou/ desistiu  13= Perdida de cartão  14= vergonha/ discriminação  15= Outro (especifique) \|__\|__\|__\|__\|__\|__\|__\|__\|__\|__\|__\|  88= não sabe |  |  |  |  |  |
|  | **Quem são os diferentes acompanhantes da criança para as consultas TARV? (assinale todas aplicáveis)**  **1=** Mãe  **2=** Pai  **3=** Avó/ avô  **4=** Irmão/ irmã  **5=** Tio/ tia  **6=** Primo/ prima  **7=** Ninhem  **8=** Outro não familiar \|__\|__\|__\|__\|__\|__\|__\|__\|__\|__\|__\|__\|__\|__\|__\| |  |  |  |  |  |
|  | **Alguém levanta habitualmente os ARV para a criança na farmácia?**  **1=** Sim  **2=** Não  **88=** Não sabe  **28.1. Se sim, quem? (assinale todas aplicáveis)**  **1=** A própria criança  **2=** Mãe  **3=** Pai  **4=** Avó/ avô  **5=** Irmão/ irmã  **6=** Tio/ tia  **7=** Primo/ prima  **8=** Outro não familiar \|__\|__\|__\|__\|__\|__\|__\|__\|__\|__\|__\|__\|__\|__\|__\|  **88=** Não sabe |  |  |  |  |  |
|  | **Por quanto tempo esteve sem tomar os comprimidos contra o HIV (TARV)?**    1= 1-3 meses  2= 3-6 meses  3= 6-12 meses  4= > 1 ano  5= Não interrompeu o tratamento  88= Não sabe  29.1 Se interrompeu TARV, qual foi o motivo de interrupção?  **1=** Mudança de residência do cuidador primário  **2=** Doença do cuidador primário  **3=** Esqueceu  **4=** Cuidador primário sem dispensa no trabalho  **5= Sem medicamentos na farmácia do hospital**  **6=** Outro \|__\|__\|__\|__\|__\|__\|__\|__\|__\|__\|__\|__\|__\|__\|__\|__\|__\|__\|__\|__\|__\|__\|__\|__\|__\|__\|__\|  \|__\|__\|__\|__\|__\|__\|__\|__\|__\|__\|__\|__\|__\|__\|__\|__\|__\|__\|__\|__\|__\|__\|__\|__\|__\|__\|__\| |  |  |  |  |  |
|  | **Alguma vez a criança procurou assistência clinica por encontrar se doente desde o diagnóstico de HIV?**  **1=** Sim  **2=** Não  **88=** Não sabe |  |  |  |  |  |
|  | **Esteve internado alguma vez desde o diagnóstico de HIV?**  **1=** Sim , na Manhiça  **2= Sim, no Hospital** \|__\|__\|__\|__\|__\|__\|__\|__\|__\|__\|__\|__\|__\|__\|__\|__  **3=** Não  **88=** Não quer responder  31.1. **Se sim, quantas vezes esteve internado**? \|__\|__\| **88=** Não sabe |  |  |  |  |  |
|  | **Se fosse possível, o cuidador primário gostaria de se poder comunicar com Hospital Distrital da Manhiça por sms?**  **1=** Sim  **2=** Não  **88=** Não sabe |  |  |  |  |  |
| **TFACTORES SOCIAIS** | |  |  |  |  |  |
|  | **A criança frequenta creche ou escola?**  **1=** Sim, sempre  **2=** Não sempre  **3=** Não  **88=** Não sabe  33.1. Se sim à que classe assiste a criança? \|__\|__\| classe |  |  |  |  |  |
|  | **A criança tem o estado vacinal actualizado? (comprovar se o acompanhante tiver o cartão de saúde)**  **1=** Sim, todas  **2=** Sim, algumas vezes  **3=** Não  **88=** Não sabe |  |  |  |  |  |
