# Supplementary material for: The impact of the caregiver mobility on child HIV care in the Manhiça District, Southern Mozambique: A clinical based study
Source: PLoS One. 2021 Dec 16;16(12):e0261356. doi: 10.1371/journal.pone.0261356 (PMC8675651; doi:10.1371/journal.pone.0261356)
Supplement: S4 File — (DOCX) [file pone.0261356.s004.docx]

|  | ***To be filled in for the child's companion at the ART appointment.*** |  |  |  |  |  |
| --- | --- | --- | --- | --- | --- | --- |
|  | **SOCIO-DEMOGRAPHIC INFORMATION** |  |  |  |  |  |
|  | **study number DIAC** \|__\|__\|__\|__\| |  |  |  |  |  |
|  | **Does the child lives in the District of Manhiça, currently?**  **1=** Yes  **2=** No |  |  |  |  |  |
|  | **If the participant lives in the District of Manhiça, where?**  **1=** Vila da Manhiça **5=** Maragra **9=** Palmeira/Nwamatibjana  **2=** Xinavane **6=** Maluana **10=** Taninga  **3=** Munguini **7=** Calanga **11=** 3 Fevereiro  **4=** Ilha Josina **8=** Xibukutsu **12=** Malavel  **14=** Other \|__\|__\|__\|__\|__\|__\|__\|__\|__\|__\|__\| |  |  |  |  |  |
|  | **With whom does the child usually live? (check all applicable)**  **1=** Mother  **2=** Father  **3=** Grandmother /grandfather  **4=** Brother/ sister  **5=** Uncle aunt  **6=** Cousin  **7=** other unfamiliar \|__\|__\|__\|__\|__\|__\|__\|__\|__\|__\|__\|__\|__\|__\|__\| |  | | | |  |
|  | **Who is the child's primary caregiver?**  **1=** Mother  **2=** Father  **3=** Grandmother /grandfather  **4=** Brother/ sister  **5=** Uncle aunt  **6=** Cousin  **7=** other unfamiliar \|__\|__\|__\|__\|__\|__\|__\|__\|__\|__\|__\|__\|__\|__\|__\| |  | | | |  |
|  | **Who is the child's companion in this consultation?**  **1=** Mother  **2=** Father  **3=** Grandmother /grandfather  **4=** Brother/ sister  **5=** Uncle aunt  **6=** Cousin  **7=** other unfamiliar \|__\|__\|__\|__\|__\|__\|__\|__\|__\|__\|__\|__\|__\|__\|__\| |  | | | |  |
|  | **Is the child's companion in this consultation a primary caregiver or an accompanying caregiver?**  **1= Primary caregiver**  **2= Accompanying caregiver** |  | | | |  |
|  | **Where does the child's mother live?**  1= In the same household as the child  2= In another household within the district of Manhiça  3= In another household outside the district of Manhiça  4= In another country  8.1. If doesn't live with the child, why? (Tick all applicable)  1= passed away  2= Migration or frequent travel  3= Work  4= Disease  5= family separation  6= Abandonment  7= Don't want to answer  88= Don’t know  9= Other \|__\|__\|__\|__\|__\|__\|__\|__\|__\|__\|__\|__\|__\|__\|__\| |  | | | |  |
|  | **Where does the child's father live?**  1= In the same household as the child  2= In another household within the district of Manhiça  3= In another household outside the district of Manhiça  4= In another country  9.1. If doesn't live with the child, why? (Tick all applicable)  1= passed away  2= Migration or frequent travel  3= Work  4= Disease  5= family separation  6= Abandonment  7= Don't want to answer  88= Don’t know  9= Other \|__\|__\|__\|__\|__\|__\|__\|__\|__\|__\|__\|__\|__\|__\|__\| |  | | | |  |
|  | **Full name** \|__\|__\|__\|__\|__\|__\|__\|__\|__\|__\|__\|__\|__\|__\|__\|__\|  \|__\|__\|__\|__\|__\|__\|__\|__\|__\|__\|__\|__\|__\|__\|__\|__\|  \|__\|__\|__\|__\|__\|__\|__\|__\|__\|__\|__\|__\|__\|__\|__\|__\| |  | | | |  |
|  | **Father's name** \|__\|__\|__\|__\|__\|__\|__\|__\|__\|__\|__\|__\|__\|__\|__\|__\|  \|__\|__\|__\|__\|__\|__\|__\|__\|__\|__\|__\|__\|__\|__\|__\|__\|  \|__\|__\|__\|__\|__\|__\|__\|__\|__\|__\|__\|__\|__\|__\|__\|__\| |  | | | | |
|  | **Mother's name** \|__\|__\|__\|__\|__\|__\|__\|__\|__\|__\|__\|__\|__\|__\|__\|__\|  \|__\|__\|__\|__\|__\|__\|__\|__\|__\|__\|__\|__\|__\|__\|__\|__\|  \|__\|__\|__\|__\|__\|__\|__\|__\|__\|__\|__\|__\|__\|__\|__\|__\| |  | | | | |
|  | **Aggregate Head Name** \|__\|__\|__\|__\|__\|__\|__\|__\|__\|__\|__\|__\|__\|__\|__\|__\|  \|__\|__\|__\|__\|__\|__\|__\|__\|__\|__\|__\|__\|__\|__\|__\|__\|  \|__\|__\|__\|__\|__\|__\|__\|__\|__\|__\|__\|__\|__\|__\|__\|__\| |  | | | | |
|  | **Neighborhood in which you live** \|__\|__\|__\|__\|__\|__\|__\|__\|__\|__\|__\|__\|__\|__\|__\|__\| |  |  |  |  |  |
|  | **Date of birth** \|__\|__\| - \|__\|__\|__\| - \|__\|__\|__\|__\| |  | |  |  |  |
|  | **Perm_id** \|__\|__\|__\|__\|- \|__\|__\|__\|-\|__\|__\| |  | | |  |  |
|  | **N. de HDD 02/009/**\|__\|__\|/\|__\|__\|__\|__\| |  | | |  |  |
|  | **Educational level of the primary caregiver:**  **1= Did not study**  **2= Below 5th grade**  **3= 5th grade**  **4= 7th grade**  **5= 10th grade**  **6= 12th grade**  **7= Elementary technical education**  **8= Basic technical education**  **9= high school technical education**  **10= Bachelor**  **11= Degree**  **12= Master**  **13= PhD**  **14= Other**  **88= Don't know** |  | | |  |  |
|  | **What is the main source of income of the household where the participant lives in the district of Manhiça?**  **1= Peasant**  **2= Salaried**  **3= No fixed salary**  **4= Don't know or don't want to answer**  **5= Other \|__\|__\|__\|__\|__\|__\|__\|__\|__\|__\|__\|** |  | | |  |  |
|  | **Child's primary caregiver job:**  **1=Administrative/employee/work for the state**  **2=Own/boss business**  **3=Agriculture**  **4=Industry**  **5=Mine**  **6=Seller**  **7=Domestic work**  **8=Not working**  **9=Other**  **88= Don't know** |  | | |  |  |
|  | **Main caregiver's religion:**  **1= Catholic**  **2= Protestant/Anglican**  **3= undetermined Christian**  **4= Islamic**  **5= Hindus**  **6= Zion/ Zion**  **7= Animists**  **8= Evangelical / Pentecostal**  **9= Atheist**  **10= Other (please specify) \|__\|__\|__\|__\|__\|__\|__\|__\|__\|__\|__\|**  **88= Don't know**  **99= Refusal** |  | | |  |  |
|  | **How many cell phones does the primary caregiver have?**  **1=None**  **2= 1**  **3= 2-4**  **4=>5**  **88= Don't know** |  | | |  |  |
| **HIV HISTORY** | | |  |  |  |  |
|  | **How long has the child been diagnosed with HIV??**  **1= <3 months 2= 3-12 months 3=1-5 years 4= > 5 years 88= Don't know**  23.2. **Where was the child diagnosed with HIV??**  **1=Manhiça District Hospital**  **2= Another Health Post within Manhiça district**  **3= Another place within Mozambique**  **4= In another country**  **88= Don't know**  23.3 **If diagnosed in another country, in which?**  **1= South Africa**  **2= Swaziland**  **3= Lesotho**  **4= Zimbabwe**  **5= Tanzania**  **6= Botswana**  **7=Other** \|__\|__\|__\|__\|__\|__\|__\|__\|__\|__\|__\|__\|__\|__\|__\|__\|  88= **Don't know** | |  |  |  |  |
|  | **How long ago did the child start ART?**  **1= <3 months**  **2= 3-12 months**  **3=1-5 years**  **4= > 5 years**  **88= Don't know** |  |  |  |  |  |
|  | **How often does the child attend HIV follow-up appointments?**  **1=Weekly**  **2=Monthly**  **3=From 3/3 months**  **4=6/6 months**  **5=Once a year**  **88=Do not know** |  |  |  |  |  |
|  | **How long was the child without going to the appointments HIV follow-up in the last year?**  **1=1-3 months**  **2=3-6 months**  **3=6-12 months**  **4=> 1 year**  **5=Did not interrupt follow-up in HIV consultations**  **88=Do not know**  **26.1 If you interrupted follow-up, please indicate the reason (check all applicable)** |  |  |  |  |  |
|  |  |  |  |  |  |  |
|  | **The child had to wait for the caregiver's authorization primary for starting ART?**  **1= Yes, from the father months 2= 3-12 months 3=1-5 years 4= > 5 years 88= Don't know**  **2= Yes, from the mother**  **3= Yes, from another family member**  **4= No**  **88= Don't know** |  |  |  |  |  |
|  | **Since the child started follow-up in ART appointments, how many times has the primary caregiver moved or traveled?**  **1= 1 time**  **2= 2 times**  **3= 3-5 times**  **4= Once a month**  **5= Once a week**  **6= Other** \|__\|__\|__\|__\|__\|__\|__\|__\|__\|__\|__\|__\|__\|__\|__\|__\|  **88= Don't know** |  |  |  |  |  |
|  | How long has the child been without HIV follow-up appointments?  1= 1-3 months  2= 3-6 months  3= 6-12 months  4= > 1 year  5= Did not interrupt follow-up in HIV consultations  88= Don't know  39.1. If the child interrupted follow-up, please indicate the reason (check all applicable)  1= Forgot  2= Did not have a child transfer document  3= Didn't know where to go  4= The primary caregiver was sick / admitted to the hospital  5= The child was sick / admitted to the hospital  6= unavailability per job  7= Transport problems  8= Absence of the health professional in the service  9= poor service  10= Side effects of treatment  11= Is in traditional treatment  12= Abandoned / gave up  13= Lost card  14= shame/ discrimination  15= Other (specify) \|__\|__\|__\|__\|__\|__\|__\|__\|__\|__\|__\|  88= Don't know |  |  |  |  |  |
|  | Who are the different caregivers of the child for ART appointments (Tick all applicable)  **1=** Mother  **2=** Father  **3=** Grandmother /grandfather  **4**= Brother/ sister  5= Uncle aunt  6= Cousin  7= None  8. Other unfamiliar |  |  |  |  |  |
|  | **After the primary caregiver moved or traveled, the child continued to come to HIV follow-up appointments Manhica District Hospital?**  1=Yes, monthly  2=Yes, every 2 months  3= Yes every 3 months  4=Yes, every 6 months  5= Once a year  6= No |  |  |  |  |  |
|  | **If the child also moved or traveled, did he/she take or ask for a transfer guide to the HIV follow-up appointments at the place of destination??**  **1=Yes**  **2=No**  **3= Not applicable, the child has not traveled**  **88= Don't know**  42.1. If the transfer slip was not made, what was the reason??  1= Did not inform about change of residence  2= Requested but not accepted  3= Other \|__\|__\|__\|__\|__\|__\|__\|__\|__\|__\|__\|__\|__\|__\|__\|  88= Don't know |  |  |  |  |  |
|  | **If the child has moved or has traveled, was it ever the HIV consultations at the place of destination?**  **1= Yes, ever**  **2= Yes, regularly**  **3= No**  **88= Don't know** |  |  |  |  |  |
|  | **If the child moved or traveled, did he/she have access to ARVs in the place of destination?**  **1=Yes**  **2=No**  **88= Don't know**  **44.1 If not, what was the reason?**  **1= Did not seek clinical care**  **2= Didn't know I could be followed at the destination**  **3= Follow-up was not accepted at the health unit where he sought**  **4= There was no ARV**  **5= Other \|__\|__\|__\|__\|__\|__\|__\|__\|__\|__\|__\|__\|__\|__\|__\|__\|__\|__\|__\|__\|__\|__\|__\|__\|__\|__\|**  **\|__\|__\|__\|__\|__\|__\|__\|__\|__\|__\|__\|__\|__\|__\|__\|__\|__\|__\|__\|__\|__\|__\|__\|__\|__\|__\|**  **88= Don't know**  **44.2 If so, how did you get access to ARVs at the destination?**  **1= Sent by family/acquaintance**  **2= Local pharmacy**  **3= Bought at the market or store**  **4= Other \|__\|__\|__\|__\|__\|__\|__\|__\|__\|__\|__\|__\|__\|__\|__\|__\|__\|__\|__\|__\|__\|__\|__\|__\|__\|__\|__\|** |  |  |  |  |  |
|  | **During your absence from the primary caregiver, did anyone pick up ARVs for the child at the Manhica District Hospital pharmacy last year?**  **1=Yes**  **2=No**  **3= Do not want to answer**  **88= Don't know**  **45.2 If someone raised for the child, how did the ARVs arrive at the place of destination?**  **1 = Post office**  **2 = Someone brings to the place of destination**  **3 = The participant takes it from Manhica, when he returns**  **4 = Other**  **45.3 Did the ARVs arrive on time?**  **1 = Ever**  **2 = Most of the time**  **3 =Sometimes (like half/half)**  **4= No, they never arrived in time**  **88 = Don't know**  **45.4 What used to do when the child ran out of ARV shipped from Manhiça?**  **1 = Looked for ARVs at the destination's health unit**  **2 = Bought the ARVs at the destination**  **3 = Took fewer pills until the ARVs arrived**  **4 = Did not receive treatment until ARVs arrived**  **5 = Other**  **6 = Do not want to answer** |  |  |  |  |  |
|  | **Has the child ever been to the hospital for being sick in the last year?**  **1=Yes**  **2=No**  **3=Don't know** |  |  |  |  |  |
|  | **If the child has also moved or traveled, has he/she ever sought medical care because he/she was sick in the place of destination in the last year?**  **1=Yes**  **2=No**  **3=Not applicable**  **4= Don't know** If YES where? **1= Emergency/Relief Bank**  **2= ART consultation**  **3= Pharmacy**  **4= Screening**  **5= Other \|__\|__\|__\|__\|__\|__\|__\|__\|__\|__\|__\|__\|__\|__\|__\|**  **88= Don't know** |  |  |  |  |  |
|  | **Has the child ever been sick when the primary caregiver was away last year?**  **1=Yes**  **2=No** |  |  |  |  |  |
|  | **Has the child ever been hospitalized in the last year?**  **1=Yes, in Manhica**  **2=Yes, at the destination**  **3=Yes, in another place**  **4=No**  **5=Do not want to answer**  **49.1. If so, how many times has she been hospitalized? \|__\|__\|__\|** |  |  |  |  |  |
|  | **Has the child ever been hospitalized when the primary caregiver was away last year?**  **1=Yes**  **2=No**  **88= Don't know** |  |  |  |  |  |
|  | **How long has the child been without taking HIV pills (ART) in the last year?**  **0=Less than 1 month**  **1=1-3 months**  **2=3-6 months**  **3=6-12 months**  **4=> 1 year**  **5=Did not stop the treatment**  **88=Do not know**  **51.1 If you interrupted ART, what was the reason for the interruption?**  **1= Change of residence of primary caregiver**  **2= Primary caregiver disease**  **3= Forgot**  **4= Primary caregiver without work time off**  **5= No medication at the hospital pharmacy**  **6= Other (especificar)** \|__\|__\|__\|__\|__\|__\|__\|__\|__\|__\|__\|__\|__\|__\|__\|__\|__\|__\|__\|__\|__\|__\|__\|  \|__\|__\|__\|__\|__\|__\|__\|__\|__\|__\|__\|__\|__\|__\|__\|__\|__\|__\|__\|__\|__\|__\|__\|__\|__\|__\|__\|__\|__\| |  |  |  |  |  |
|  | **If the child has traveled, has he/she ever had CD4 or viral load follow-up at the place of destination?**  **1=Yes**  **2=No**  **3= not applicable**  **88= Don't know** |  |  |  |  |  |
|  | **If possible, the primary caregiver would like to be able to communicate with the Manhica District Hospital by SMS at the place of destination**  **1=Yes**  **2=No**  **3=Not applicable**  **4= Don't know** |  |  |  |  |  |
|  | **If possible, would you like to be able to collect a larger amount of ARV for the child at the Manhica District Hospital to take at the destination?**  **1=Yes**  **2=No**  **3=Don't know**  **54.1. If yes, how much would you like to take for what period?**  **1=Only for 3 months**  **2=3-6 months**  **3=Up to 6 months**  **4=Other (in months)** |  |  |  |  |  |
|  | **Which of the following difficulties did you encounter when you returned to the child's HIV follow-up appointments at the Manhica District Hospital?**  **1=Too much waiting time**  **2=Bad service because I didn't have a card**  **3=Poor service because there was no clinical process**  **4=Poor service by staff**  **5=Did not find the location of the consultation**  **6=Others (especificar)** \|__\|__\|__\|__\|__\|__\|__\|__\|__\|__\|__\|__\|__\|__\|__\|__\|__\|__\|__\|__\|__\|__\|__\|  **7=Had no difficulties** |  |  |  |  |  |
| **SOCIAL FACTORS** | |  |  |  |  |  |
|  | **Does the child attend daycare or school?**  **1= Yes, regularly**  **2= Yes, a few days**  **3= No**  **88= Don't know**  **56.1. If Yes, do you keep attending daycare or school when your caregiver is away?**  **1=Yes**  **2=No**  **88= Don't know**  **56.2. If so, which level does the child attend? \|__\|__\|** |  |  |  |  |  |
|  | **Does the child have up-to-date vaccination status? (check if the companion has the health card)**  **1= Yes, all**  **2= Yes, sometimes**  **3= No**  **88= Don't know** |  |  |  |  |  |
