# Supplementary material for: The impact of the caregiver mobility on child HIV care in the Manhiça District, Southern Mozambique: A clinical based study
Source: PLoS One. 2021 Dec 16;16(12):e0261356. doi: 10.1371/journal.pone.0261356 (PMC8675651; doi:10.1371/journal.pone.0261356)
Supplement: Attachment — Submitted filename: Response to reviewers PONE-D-21-13785 November 2021.pdf [file pone.0261356.s006.pdf]

To the Editor of PLOS ONE

Maputo, 15th November, 2021

Thank you very much for the helpful review of our manuscript. Please find below our responses to all comments raised by the editors and the reviewers in regards to manuscript PONE-D-21-13785, entitled "The impact of the caregiver mobility on child HIV care in the Manhiça District, Southern Mozambique: a clinical based study".

**EDITOR'S COMMENTS:**

1. Please ensure that your manuscript meets PLOS ONE's style requirements, including those for file naming.
   Answer: We have revised the manuscript in order to meet PLOS ONE's style requirements.

2. Please include additional information regarding the survey or questionnaire used in the study and ensure that you have provided sufficient details that others could replicate the analyses. For instance, if you developed a questionnaire as part of this study and it is not under a copyright more restrictive than CC-BY, please include a copy, in both the original language and English, as Supporting Information. If the original language is written in non-Latin characters, for example Amharic, Chinese, or Korean, please use a file format that ensures these characters are visible.
   Answer: All information regarding the questionnaire used in the study appear in the "Materials and methods section". Two questionnaires were developed in Portuguese, the Mozambican official language (original language) as part of the study and it is not under a copyright more restrictive than CC-BY. The questionnaires have been translated to English and both uploaded together with original ones.

3. Please state whether you validated the questionnaire prior to testing on study participants. Please provide details regarding the validation group within the methods section.
   Answer: The questionnaire has not been validated. In the week prior to the start of the study, the questionnaires and study procedures were piloted.

4. We note that you have indicated that data from this study are available upon request. PLOS only allows data to be available upon request if there are legal or ethical restrictions on sharing data publicly. For more information on unacceptable data access restrictions, please see http://journals.plos.org/plosone/s/data-availability#loc-unacceptable-data-access-restrictions.

   Answer: We have now indicated that data from this study are fully available without restriction.

   In your revised cover letter, please address the following prompts:

a) If there are ethical or legal restrictions on sharing a de-identified data set, please explain them in detail (e.g., data contain potentially sensitive information, data are owned by a third-party organization, etc.) and who has imposed them (e.g., an ethics committee). Please also provide contact information for a data access committee, ethics committee, or other institutional body to which data requests may be sent.

   Answer: We have addressed in the cover letter that there are no ethical or legal restrictions on sharing a de-identified data set.

b) If there are no restrictions, please upload the minimal anonymized data set necessary to replicate your study findings as either Supporting Information files or to a stable, public repository and provide us with the relevant URLs, DOIs, or accession numbers. For a list of acceptable repositories, please see http://journals.plos.org/plosone/s/data-availability#loc-recommended-repositories.

   Answer: We have uploaded the minimal anonymized data set necessary to replicate our study findings.

5. PLOS requires an ORCID iD for the corresponding author in Editorial Manager on papers submitted after December 6th, 2016. Please ensure that you have an ORCID iD and that it is validated in Editorial Manager. To do this, go to 'Update my Information' (in the upper left-hand corner of the main menu), and click on the Fetch/Validate link next to the ORCID field. This will take you to the ORCID site and allow you to create a new iD or authenticate a pre-existing iD in Editorial Manager. Please see the following video for instructions on linking an ORCID iD to your Editorial Manager account: https://www.youtube.com/watch?v=_xcclfuvtxQ.

   Answer: The ORCID iD for the corresponding author has been updated in Editorial Manager.

6. We note that you have included the phrase "data not shown" in your manuscript. Unfortunately, this does not meet our data sharing requirements. PLOS does not permit references to inaccessible

data. We require that authors provide all relevant data within the paper, Supporting Information files, or in an acceptable, public repository. Please add a citation to support this phrase or upload the data that corresponds with these findings to a stable repository (such as Fig share or Dryad) and provide and URLs, DOIs, or accession numbers that may be used to access these data. Or, if the data are not a core part of the research being presented in your study, we ask that you remove the phrase that refers to these data.

Answer: We have removed the phrase "data not shown". In fact we wanted to mention that this information only appears in the text paper but not in tables.

7. Please include a copy of Table 3 which you refer to in your text on page 15.

   Answer: A copy of the Table 3 is include in page 16, however, there was no mention of table 3 in the text. We have now corrected it, by mentioning table 3 in text line 276 (Revised Manuscript with NO Track Changes).

8. Please review your reference list to ensure that it is complete and correct. If you have cited papers that have been retracted, please include the rationale for doing so in the manuscript text, or remove these references and replace them with relevant current references. Any changes to the reference list should be mentioned in the rebuttal letter that accompanies your revised manuscript. If you need to cite a retracted article, indicate the article's retracted status in the References list and also include a citation and full reference for the retraction notice.

   Answer: The reference list was reviewed and there is no citation of retracted papers. Thus no change have been made to the reference list.

**REVIEWER #2:**

1. This is an interesting study addressing a topic with clear social and scientific value. The motivation for this study is well presented and the objectives are articulated well. The impact of the results is limited by the relatively small sample size of the study. Although the chosen period of the study is justified by the author's assumption that hospital visits increase two to three fold during this time, it is not clear whether secular trends could affect this. Therefore a longer period than the 3 month period would have been useful to account for this. There is need to provide an estimate of the denominator figure for Children Living with HIV in the Manhica area and the number receiving care at the Manhica district hospital to give the reader perspective.

   Answer: We have included in Materials and Methods - Study setting section a phrase regarding the denominator figure for Children Living with HIV in the Manhiça area and the number

receiving care at the Manhiça district hospital according to the local health authority's annual report in order to give the reader perspective (in text line 109 in the Revised Manuscript with NO Track Changes).

2. Although the results presented (both in the abstract and the main body of the manuscript) highlight non-significant association (thus negative results regarding these associations), the conclusion has ignored the implication of these. Similarly the lack of association is not discussed in the discussion section. If these were hypothesized a priori (and thus included in the conditional logistic model), they need to be fully discussed in light of the literature.

   Answer: The lack of association between caregiver's mobility child continuations in HIV care had not been hypothesized a priori. In order to highlight the non-significant association found in the study, we have included the phrase "…..The caregiver mobility was not found to significantly affect child's retention on ART…" in the two conclusions sections (lines 48 and 372) and the phrase "However, our results show that none of the mobility pattern impacted on the child HIV care……" in third paragraph of the discussion section line 309.

3. The data analysis plan (statistical methods) refers to use of parametric tests for normal continuous variables. However these tests are not specified and a review of the results show categorical variables and non-normally distributed variables. The write up in the statistical methods section limits the analysis of categorical data to the Chi-squared test (omits the appropriateness of Fisher's exact test) even though the footnote in Table 1 indicates that Fisher's exact test was used where appropriate.
   Answer: We corrected and updated the information about the statistical tests in the Statistical methods section line 175 and also in the footnote of table 1 line 216.

4. Details about the estimated sample size as well as assumptions used are missing.
   Answer: Details about the estimated sample size as well as assumptions used are now presented in the Sample size calculation section line 144.

We have uploaded the following documents:

Revised Manuscript with Track Changes.

Revised Manuscript with no Track Changes.

Questionnaires in Portuguese and English language

De-identified data set

Kind regards,

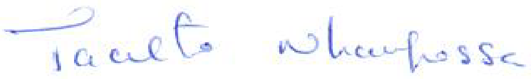

Tacilta Nhampossa
